# T Cell-Mediated Chronic Inflammatory Diseases Are Candidates for Therapeutic Tolerance Induction with Heat Shock Proteins

**DOI:** 10.3389/fimmu.2017.01408

**Published:** 2017-10-26

**Authors:** Ariana Barbera Betancourt, Qingkang Lyu, Femke Broere, Alice Sijts, Victor P. M. G. Rutten, Willem van Eden

**Affiliations:** ^1^Faculty of Veterinary Medicine, Department of Infectious Diseases and Immunology, Utrecht University, Utrecht, Netherlands

**Keywords:** heat shock proteins, tolerance, T regulatory cells, rheumatoid arthritis, inflammatory eye diseases, diabetes mellitus, type 1

## Abstract

Failing immunological tolerance for critical self-antigens is the problem underlying most chronic inflammatory diseases of humans. Despite the success of novel immunosuppressive biological drugs, the so-called biologics, in the treatment of diseases such rheumatoid arthritis (RA) and type 1 diabetes, none of these approaches does lead to a permanent state of medicine free disease remission. Therefore, there is a need for therapies that restore physiological mechanisms of self-tolerance. Heat shock proteins (HSPs) have shown disease suppressive activities in many models of experimental autoimmune diseases through the induction of regulatory T cells (Tregs). Also in first clinical trials with HSP-based peptides in RA and diabetes, the induction of Tregs was noted. Due to their exceptionally high degree of evolutionary conservation, HSP protein sequences (peptides) are shared between the microbiota-associated bacterial species and the self-HSP in the tissues. Therefore, Treg mechanisms, such as those induced and maintained by gut mucosal tolerance for the microbiota, can play a role by targeting the more conserved HSP peptide sequences in the inflamed tissues. In addition, the stress upregulated presence of HSP in these tissues may well assist the targeting of the HSP induced Treg specifically to the sites of inflammation.

In many cases, chronic inflammatory diseases are autoimmune diseases that are caused by a loss of tolerance to self-antigens due to inappropriate activation of the immune system. Collectively, autoimmune diseases affect 4–5% of the population, being females affected with a higher incidence than males (3:1 ratio) (1).

Genome-wide association studies have underscored the genetic association of the major histocompatibility complex (MHC) region with autoimmune diseases, in which case various predisposing alleles have been found (2, 3). The main function of MHC molecules is to present processed peptides for the recognition of antigen-specific T cells. And such T cells have the capacity to damage healthy tissues when they are not tightly controlled. The exact mechanisms triggering autoimmune diseases are unknown, but the presence of pro-inflammatory T cells in target organs as well as the strong link with MHC loci highlights the important role for adaptive immune responses in their development. The most accepted hypothesis proposes that for the initiation of an autoimmune disease, an immune response with pro-inflammatory characteristics needs to be directed against specific tissue antigens in genetically susceptible individuals. Regulatory mechanisms exist in the periphery to control such effector responses to avoid excessive tissue damage (4). Mechanisms include the following: regulatory T cells (Tregs), direct inactivation of effector T (Teff) cells by induction of anergy or apoptosis and activities mediated by tolerogenic antigen-presenting cells (APCs). However, there is an increasing understanding that pro-inflammatory responses directed to self-antigens become chronic in autoimmune diseases because regulatory mechanisms fail to control them.

## Peripheral Tolerance Mechanisms

### CD4^+^CD25^high^Foxp3^+^

**Figure 1:**
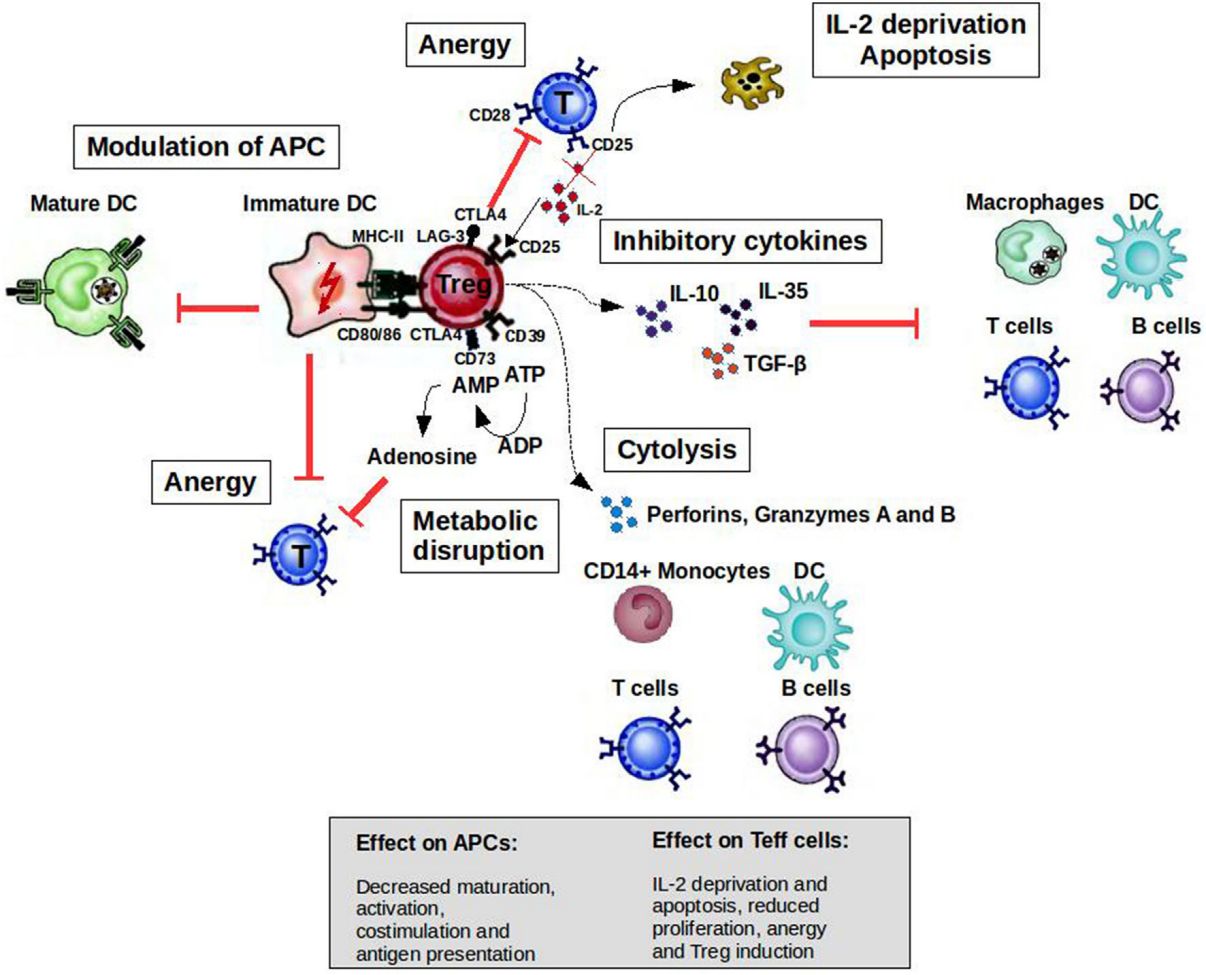
Mechanisms of suppression by Treg cells to control immune responses. A broad range of molecular mechanisms contribute to the suppressive function of Tregs. Mechanisms include the following: apoptosis/cytolysis (IL-2 deprivation, granzyme A/B, perforins); antigen-presenting cell (APC) modulation (CTLA4, LAG-3); inhibitory cytokines (IL-10, IL-35, and TGF-β); and metabolic disruption (CD73/39 and ATP/adenosine mechanism). Abbreviations: CTLA4, cytotoxic T lymphocyte-associated antigen 4; DC, dendritic cell; CD, cluster of differentiation; IL, interleukin; Treg cell, regulatory T cell; LAG-3, lymphocyte activation gene 3; TGF, transforming growth factor; MHC, major histocompatibility complex.

CD4^+^CD25^high^FoxP3^+^ Tregs can prevent autoimmune diseases by maintaining the tolerance to self-antigens. FoxP3 constitutes the most specific marker for these cells and is to some extent indispensable to develop a Treg phenotype and for their suppressive function (5). The development of autoimmune diseases when CD4^+^CD25^+^ cells are depleted in normal rodents or when rodents and humans have mutated FoxP3 genes highlights the role of Tregs in the prevention of such diseases (6, 7). As illustrated in Figure 1, when activated by their cognate antigen, Treg cells display a broad range of suppressive mechanisms, which endow them with the ability to control immune responses. The potential of controlling T- and B-cell responses with different specificities as well as the modulation of the maturation status of APCs by Tregs makes them attractive targets for the development of therapeutic strategies. Apart from CD4^+^CD25^high^Foxp3^+^ Tregs, there are several subsets of CD8^+^ T cells that are able to downregulate CD4^+^ T-cell effector responses by different mechanisms including the induction of anergy in APCs and T cells as well as the secretion of anti-inflammatory cytokines (8). CD8^+^CD28^−^Foxp3^+^ Treg cells are probably the subset best characterized (9). The activation of these cells is antigen specific [major histocompatibility complex (MHC)-I class-restricted], and their suppressor mechanism involves the induction of a tolerogenic phenotype in APCs by the increased expression of immunoglobulin-like transcript 3 (ILT3) and ILT4. ILT3 and ILT4 suppress the activation of nuclear factor-κB mediated by CD40, which in turn reduces the transcription of co-stimulatory molecules such as CD80 and CD86 (9–11). These tolerogenic APCs in turn promote an anergic phenotype on naive CD4^+^ and CD8^+^ T cells, which could acquire similar regulatory functions spreading the induction of tolerance (10).

However, some studies have shown that patients with autoimmune diseases have less effective or fewer CD4^+^CD25^high^Foxp3^+^ Treg cells compared with healthy individuals [reviewed in Ref. (12)]. Numbers and/or function of CD8^+^ Tregs have been also found to be defective in animal models of autoimmunity and in patients (13). Defects in the capacity of Teff cells to be controlled by Tregs have also been found in the context of autoimmune diseases (12). Collectively, these findings suggest that Treg malfunction might be a factor promoting the development or chronicity of autoimmune diseases. Therefore, approaches to expand regulatory populations in autoimmune diseases have therapeutic potential (14, 15).

### Anergy

T cells are activated when their T-cell receptors (TCRs) recognize antigenic peptides presented by MHC molecules expressed on the surface of APCs. Secondary signals like the one provided by CD28 expressed by T cells and B7.1 (CD80) or B7.2 (CD86) expressed by APCs are essential to initiate IL-2 production and T-cell proliferation. However, the activation of T cells without second signals induces a state of anergy where these clones are not able to respond to antigenic stimulus because they cannot produce IL-2. Cytotoxic T lymphocyte-associated antigen-4 (CTLA4) is a cell surface molecule related to CD28 that has the ability to block CD28-dependent T cell activation (16). The critical role of this molecule in controlling T-cell activation and maintaining peripheral tolerance was supported by the development of a massive lymphoproliferative disorder and autoimmune disease being fatal by 3–4 weeks of age in CTLA-4-deficient mice (17). Activated T cells transiently increase the expression of CTLA4, which is important to limit the expansion of activated T cells during an immune response. This cell surface molecule is expressed constitutively by Tregs endowing them with the potential to control T-cell activation through CD28 blockade (Figure 1). The inhibition of the CD28-dependent T cell activation has been used as a therapeutic tool for several autoimmune diseases. The blockade of the CD28 pathway with CTLA-Ig in animal models of autoimmune diseases prevented the progression of the disease [reviewed in Ref. (18)]. Abatacept (CTLA4-Ig) has been approved by the FDA for use in rheumatoid arthritis (RA) patients with an inadequate response to one or more of the disease-modifying antirheumatic drugs.

### Apoptosis

Apoptotic cell death is another important regulatory mechanism operating in the thymus and periphery to delete self-reactive T cells or activated pathogenic T-cell clones, respectively. During the development of T cells in the thymus, clones bearing autoreactive TCRs are eliminated by apoptosis in a process known as negative selection. However, T cells with potential autoreactive receptors escape to the periphery where these clones should be kept in check by regulatory mechanisms such as Tregs, anergy, or deletion. In the periphery, activated T cells express death receptors belonging to the tumor necrosis factor (TNF) family (e.g., Fas/Fas-ligand) making them susceptible to activation-induced cell death (AICD) (19, 20). Memory T cells, Tregs, and Th2 cells are less susceptible than Th1 cells to AICD (21, 22), allowing the polarization of the immune response to protective responses (Th2/Treg) in the periphery. On the other hand, by inducing IL-2 deprivation and secreting perforins and granzymes, Tregs at the site of inflammation increase the susceptibility of Teff cells and other cells such as B cells and monocytes to cell death (23, 24).

### Tolerogenic APC

Tolerogenic APCs present antigens to T cells but since they display low numbers of co-stimulatory molecules such as CD80, CD86, and CD40, antigen presentation leads to T-cell anergy (25). Tolerogenic APCs can be induced and enhanced using different compounds such as rapamycin, corticosteroids, interleukin-10 (IL-10), and transforming growth factor beta 1 (26). Several studies have shown the therapeutic effect of tolerogenic APCs induced *ex vivo* in experimental animal models [reviewed in Ref. (27, 28)]. Treg cells can also modulate the maturation status of APCs. For example, these cells can decrease the expression of co-stimulatory molecules on APC affecting their capacity to activate T cells (29). In addition, ligation of CTLA4 to CD80 and CD86 induces APC to express an immunosuppressive molecule (indoleamine 2,3-dioxygenase), which is able to abolish T-cell activation (30, 31). Lymphocyte activation gene 3 (LAG-3) is another molecule expressed by Tregs that could affect APC function. This is a CD4 homolog with a high affinity for MHC class II molecules. The binding of LAG-3 to MHC class II induces an inhibitory signaling pathway, which leads to the inhibition of APC maturation (Figure 1) (32).

## MHC-Associated Diseases are T Cell-Mediated and Possible Targets for Induction of Heat Shock Protein (HSP)-Driven Therapeutic Tolerance

The strong link of autoimmune diseases with MHC loci and the presence of pro-inflammatory T cells in target organs highlight the important role for adaptive immune responses in their development. In such cases, therapeutic tolerance may become established through the induction of Tregs with bystander regulatory activities leading to the inhibition or modulatory skewing of these pro-inflammatory self-antigen-specific T cells. Examples of MHC-associated, primarily T-cell driven autoimmune diseases are RA, type 1 diabetes (T1D), and several eye diseases.

### Rheumatoid Arthritis

Rheumatoid arthritis is a chronic inflammatory disease characterized by joint inflammation and synovial hyperplasia, which leads to cartilage and bone destruction (33).

The HLA-DRB1 gene has been associated with the susceptibility of this disease, especially with the shared epitope (SE) coding alleles (HLA-DRB1*0401, *0404, *0405, *0408, *0101, *0102, *1402, and *1001). The SE is a five amino-acid sequence motif found in residues 70–74 of the HLA-DRβ chain that encodes a conserved positively charged residue at position 71 (34). The latter seems to guide the nature of the amino acid that can be accommodated in the P4 pocket of these HLA-DR molecules. Although the susceptibility of this disease appears to be determined genetically, the onset might depend on other factors such as environmental, epigenetic or posttranslational events factors (35).

As expected by the strong association of HLA-DRB1 and RA, CD4^+^ T cells are enriched in synovia of these patients and seem to play a critical role in the perpetuation of inflammation [reviewed in Ref. (36)]. Susceptibility to RA has also been linked to other pathways implicated in the activation of T cells, such as PTPN22, PTPN2, CTLA4, IL2RA, IL-2RB, among others [reviewed in Ref. (29)]. Specifically, a CD4^+^ T cell subset that produces IL-17, 21, 22, and TNF-α has been in the center of the attention in recent years. Emerging data have suggested that active RA might result from an imbalance between defective Tregs and pro-inflammatory Th17 cells (37–39). Nevertheless, the mechanisms governing such imbalance that could contribute to RA chronicity have remained unclear.

Tumor necrosis factor-α has been shown to be the master element of inflammation in RA (40). Consequently, the blockade of this cytokine has emerged as the main tool for its treatment. Although the exact mechanism underlying clinical effects of anti-TNF-α therapy in patients is not completely understood it is apparent that it can have an effect on other pathways associated with tolerance (41). For instance, it has been reported that the treatment with infliximab increases the percentage of CD4^+^CD25^+^ Tregs in RA patients who responded to therapy (42). Further studies showed that infliximab induced a distinct Treg population *in vitro* that could compensate the compromised Tregs detected in RA (43). Despite excellent results in patients responding to anti-TNF-α therapy, there is an increased susceptibility to serious adverse effects including: infectious diseases, malignancies and demyelination (44). In addition, only partial responses are achieved with this treatment and a continuous treatment is required.

### Diabetes Mellitus Type 1

Pancreatic β cells producing insulin are the targets for antigen-specific T cells in T1D. Epidemiologic studies suggest that the incidence of this disease is rising (45). The updated estimates of the incidence (20.04 per 100,000 per year) and prevalent cases (129,350) of T1D in children 0–14 years old in Europe for 2013 (46) reflect an increasing trend of 3–4% per annum during the past 20 years (47).

HLA-DRB1*0401-DQB1*0302 and HLA-DRB1*0301-DQB1*0201 have been associated with T1D susceptibility whereas the haplotypes HLA-DRB1*1501 and HLA-DQA1*0102-DQB1*0602 confer resistance (48). However, most people bearing the haplotypes associated with the greatest susceptibility do not develop the disease. In addition, despite the finding of islet-specific T cells in the blood of healthy individuals, one study showed that these cells secrete IL-10 instead of interferon gamma (IFN-γ) (49), indicating that regulatory mechanisms should fail to develop T1D. Indeed, there is evidence supporting that regulation is impaired in this disease, where patients seem to have a decreased Treg suppressive functionality compared with non-diabetic controls (50, 51).

The exact mechanism by which β cells are destroyed in the pancreas is not fully understood, but genetic and environmental factors appear to predispose individuals with defective regulatory mechanisms to develop the disease. Similar to other chronic inflammatory diseases, T1D onset requires CD4^+^ and CD8^+^ T cells [reviewed in Ref. (52)]. The latter has been demonstrated in experiments in which the precipitation or prevention of diabetes was achieved in the non-obese mice model by transfer or elimination of CD4^+^ or CD8^+^ T cells, respectively. Both cell types are able to infiltrate the pancreatic islets in mice and humans and are considered to be the final executors of the destruction of insulin-producing β cells (52). CD4^+^ and CD8^+^ T cells can induce the death of pancreatic β cells. However, as β cells only express HLA-class I, direct cytotoxicity can be only mediated by CD8^+^ T cells able to recognize appropriated peptides displayed on β-cell class I molecules. CD8^+^ T cells are able to kill β cells through different mechanisms including granzyme B and perforins, pro-inflammatory cytokines, and/or Fas/FasL interactions (52).

No drugs have been approved to halt the autoimmune process that causes the destruction of β cells in T1D (53). The main goals are the induction of a residual β-cell function. Different approaches to treat this disease have been used so far [reviewed in Ref. (54)]. One of the therapeutic approaches showing promise in T1D is the use of anti-CD3 monoclonal antibodies that have been shown to interfere antigen-specific T cell activation. However, after promising clinical trials (phase 1 and 2) in T1D patients with a recent onset, phase 3 trials fail to meet primary endpoints (55, 56).

### MHC-Associated Inflammatory Eye Diseases

Various studies have confirmed that eye diseases, such as idiopathic uveitis (57), birdshot retinochoroidopathy (BSR) (58), and sympathetic ophthalmia (59), have an association with MHC. Uveitis is the most common form of inflammatory eye disease and one of the leading causes of visual impairment and blindness. The association of the MHC class I molecule HLA-B27 with uveitis was first noted in 1973 (60). The precise molecular and pathogenic mechanisms behind the association between uveitis and HLA-B27 have remained unclear. HLA-B27 encompasses around 105 known subtypes (HLA-B*27:01 to HLA-B*27:106 thus far identified) that are encoded by 132 alleles (61). HLA-B27 subtypes have a varied prevalence in different races and regions of the world. HLA-B*2705 and B*2702 are the main HLA-B27 subtypes in northern Europe, whereas HLA-B*2704 and B*2706 are the most widespread subtype among Asian populations (62). A study from China found that among northern Chinese people, ankylosing spondylitis (AS) patients with B*2704 have a stronger risk of developing uveitis than those with B*2705 in Ref. (63). Conversely, a Japanese study showed that HLA-B27 anterior uveitis (AAU) patients with the B*2704 subtype seemed to be less susceptible than patients with B*2705 (64). This suggests that HLA-B*2704 and HLA-B*2705 may be the most prevalent HLA-B27 subtypes, with observed conflicting results on the role of this molecule in AAU caused by different races and regions, genetic background, or environmental factors. Remarkably, the majority of individuals who carry susceptibility conferring HLA subtypes never develop uveitis or other systemic autoimmune disease, implying that HLA-B27 is a genetically predisposing factor for uveitis but that other genetic or environmental factors contribute to the development of uveitis. HLA-B27-associated uveitis is also closely related to other systemic autoimmune syndromes, such as AS and systemic sarcoidosis. Several studies have shown that HLA-B27-positive AS patients are more susceptible to uveitis than HLA-B27-negative patients (65, 66). 20–30% of patients with sarcoidosis were affected by uveitis (67).

Except for uveitis, also specific other ocular inflammatory diseases show a strong association with HLA. BSR and idiopathic retinal vasculitis are associated with HLA-A29 (68), with HLA-A*29.01 and HLA-A*29.02 representing the most common A29 subtypes found in BSR patients (69). The HLA-A*29.01 subtype is more frequent among Asians, whereas HLA-A*29.02 is more common among Caucasians (70). In addition, Behcet’s disease (BD) is an inflammatory disease affecting multiple organs that also include a relapsing and remitting pan-uveitis, which is strongly associated with HLA-B*51 (71). HLA-B5101 is the predominant subtype associated with BD in Japanese and Iranian patients. The association of HLA-B*5108 and BD was also found in Greek and Spanish patients. A study of Israeli showed that HLA-B*52 may also be associated with BD (72).

In humans, more and more evidence reveals that cytokines produced by autoreactive Teff cell play a pivotal role in the pathogenesis of autoimmune uveitis. Early studies suggested that the imbalance of anti- and pro-inflammatory Th2 and Th1 subsets is responsible for the pathology of uveitis. However, in recent studies, emphasis was laid on Th17 and CD4^+^CD25^+^FoxP3^+^ T regulatory cells, which produce IL-17 and IL-10, respectively. The ratio of Th17/Treg was distinctly increased at the progression of uveitis in patients and in experimental autoimmune uveitis (EAU) disease models (73, 74), and imbalance of Treg cells over Th17 cells was observed at the recovery phase of EAU (73). Th1 cells play central roles in early phase of uveitis, whereas Th17 cells act in the late phase of uveitis (75), Treg and inducible Treg cells suppress both Th1 and Th17 cell responses by counterbalancing pro-inflammatory activities of these T cells. This implies that increasing the number of Treg cells may be a promising and safe way to control MHC-associated eye diseases.

## HSP and the Induction of Therapeutic Tolerance

### HSP Proteins or Peptides As Inducers of Tregs

Initial studies reported that several HSP families were able to induce both pro-inflammatory and anti-inflammatory effects. Pro-inflammatory cytokine production mediated by HSP70 appears to be linked to the activation of toll-like receptor 2 (TLR2) and TLR4 signaling pathways on innate immune cells (76, 77). Pathogen-associated molecular patterns such as lipopolysaccharide or other proteins present in recombinant HSP produced in bacteria have been suggested to be responsible for the observed pro-inflammatory effects [reviewed in Ref. (78)]. In line with this idea, HSPs often fail to induce an inflammatory effector response in highly purified preparations (79, 80). On the contrary, other studies using non-recombinant Hsp70, boiling treatments (which cause the degradation of HSP) or antibiotics have led to the conclusion that HSPs are responsible for the activation of innate immune cells as well as T cells through TLR signaling pathways (81, 82). It seems that whether these proteins have an activating or immunosuppressive role depends on several factors including their local concentration, the nature of the HSP itself (self or microbial), among others [reviewed in Ref. (83)]. In the context of autoimmune diseases, HSP proteins have been considered as target molecules involved in their pathogenesis in part because they become highly available at sites of inflammation (83). The other main reason is the high homology between species whereby microbial HSPs can active immune responses that can be cross-reactive with self-HSPs, which in theory could provoke autoimmunity. However, autoreactivity to self-HSPs has been also found in healthy individuals (84, 85), which means that these proteins are under a tight regulation network. The latter also means that autoreactivity to self-HSPs is not a synonym of autoimmunity. In fact, self-HSP reactivity appears to be a physiological mechanism for controlling the inflammatory process (86). In this regard, several studies in mice and humans support the fact that HSP, and specially conserved epitopes have the potential for attenuating rather than triggering inflammatory responses (87, 88).

The initial indication of a possible role of HSP in the induction of therapeutic tolerance was obtained in the model of adjuvant arthritis in rats. T cells collected from diseased animals were found to respond to mycobacterial HSP60 (89). When the recombinant mycobacterial HSP60 protein was used for immunizations, no arthritis was seen to develop. Interestingly, induction of adjuvant arthritis in these immunized animals appeared not to be possible anymore. Subsequent experiments revealed that the same protection against adjuvant arthritis induction was obtained by immunizing the animals with only a conserved sequence (peptide) of mycobacterial HSP60 (90). On the basis of these latter experiments, it was concluded that the conserved peptide induced T cells that were cross-reactive with self (mammalian) HSP upregulated at the site of inflammation. In various additional studies, the regulatory nature of these cross-reactive T cells was recognized, since they were producing regulatory cytokines such as IL-10.

More recent studies have shown the HSP mediated induction of T cells with regulatory potential, which showed the actual phenotypic characteristics of the currently known Tregs (91). This was among others the case for an HSP70 derived conserved mycobacterial peptide called B29. When BALB/c mice were immunized with B29, responding T cells were collected on the basis of CD25 expression and transferred into naïve syngeneic recipient mice. Subsequently, these T cells were found to inhibit disease activity after induction of arthritis and to persist in various organs, including the joints, for more than 50 days. When during this time period, the presence of these cells was interrupted by infusion of a Treg depleting antibody, disease returned, which showed the actual disease suppressive activity of these HSP70-specific Tregs. When B29-specific T cells were selected on the basis of LAG-3 expression, it sufficed to transfer only 4,000 of these T cells to fully inhibit arthritis. Several explanations are possible for the capacity of conserved microbial HSP peptides to induce Tregs. An obvious explanation can be found in the contact of the immune system with microbiota in the gut. It is known that APCs lining the gut mucosa ingest bacteria from the microbiota. This causes transport to mesenteric lymph nodes, where the derivative microbial antigens are presented to T cells, a phenomenon that must contribute to mucosal tolerance. Since ingestion of bacteria will lead to a stress response, both in bacteria and in the APC of the host, MHC molecules will be loaded with HSP fragments in this process. By these mechanisms, both microbial and the self-cross-reactive T cells will be activated. And since such events happen in the environment of the tolerance promoting mucosa, induction of peripheral Tregs seems a direct and physiological consequence. Given the evolutionary conservation of the HSP molecules present in the complete kingdom of prokaryotes, it seems unavoidable that through the repeated contacts with bacteria, the immune system develops a focus on the conserved parts of the molecules. And by this same focus on the shared sequences between bacterial and mammalian HSPs, Tregs induced by bacterial HSP may easily cross-respond to self-HSP (over-)expressed in the inflamed tissues. Herewith the regulation, which is of a bystander nature, will be targeted toward the sites of inflammation.

### Endogenous HSP-Loaded MHC Molecules

Apart from the possibility that mucosal tolerance creates a repertoire of HSP-specific Treg, there is also reason to think that HSPs are a default antigen for Tregs in the context of healthy tolerogenic APCs in the absence of co-stimulation activating microorganisms. In various studies, HSP70 has been found to belong to the most frequent cytosolic/nuclear MHC class II natural ligand sources (92). In other words, MHC elution studies have revealed that sequences of HSP70 family members are relatively often present in the proteome obtained from the antigen-binding clefts of human and mouse MHC-II molecules. And especially in the case of cell stress, such as the stress caused by inflammatory mediators, HSP70 fragments have been seen to become preferentially uploaded into MHC-II molecules [reviewed in Ref. (87)]. Dengjel et al. (93) have analyzed the sequences eluted from human B cell-derived HLA-DR4 molecules under amino-acid deprivation as the cell-stress factor. It was shown that under such conditions chaperone-mediated autophagy became operative, which led to involvement of HSP70, which is one of the molecular participants in the process of chaperone mediated autophagy. In general terms, it was seen that under stress, the presentation of peptides from intracellular and lysosomal source proteins was strongly increased on MHC-II in contrast to peptides from membrane and secreted proteins. For these reasons, it was concluded that their study illustrated a profound influence of autophagy on the class II peptide repertoire and suggested that this finding had implications for the regulation of CD4(+) T cell-mediated processes. Interestingly, also the mammalian homologs of our earlier defined HSP70-derived B29 peptide were eluted from this HLA-DR4 molecule (HLA DRB1*0401). Knowing this, it seems reasonable to think that HSPs and HSP70 family members in particular are frequently seen by Tregs in the context of MHC-II molecules. In this manner, they could well serve as a default antigen for Tregs, especially when presented by tolerogenic APC.

Given the stress-inducible nature of various HSP70 family members, we have attempted to raise the abundance of HSP70 fragments in MHC-II molecules of APCs by administering a so-called HSP co-inducing compound (94, 95). And indeed, in the experimental model of proteoglycan-induced arthritis (PGIA) in mice, we have seen such an intervention with an HSP co-inducer to lead to a T cell-mediated resistance against arthritis. The experiments were carried out with carvacrol, an essential oil obtained from Oregano plants. Initial studies *in vitro* had indicated that incubation of cells before further exposure to classical stressors, such as raised temperature or arsenite, caused a raised expression of endogenous HSP70. When given orally, carvacrol was found to lead to a raised expression of HSP70 family members in the Peyer’s patches, the lymphoid organs of the gut. In addition, when analyzing the T cell responses to HSP70, it was found that oral carvacrol had raised the frequency of HSP70-specific T cells and that such cells showed to an enhanced degree the CD4^+^CD25^+^Foxp3^+^ phenotype of Tregs. Transfer of these cells into naïve recipients inhibited subsequently induced PGIA. Thus, the experiments with carvacrol have indicated that for the purpose of generating therapeutic tolerance, the co-induction of HSPs, even by dietary measures, may be a possible and attractive strategy.

### How to Induce HSP-Based Therapeutic Tolerance in T Cell-Mediated Autoimmune Diseases

Effective interventions in animal models are usually based on disease inhibition by administering therapeutics before disease induction. In other words, effective interventions are mostly preventive and not therapeutic. Successful therapeutic interventions, when overt disease has been established, are notoriously difficult to reach. The reason for this is that established inflammation is known to cause a relative resistance to therapy, among others by a supposed resistance of effector cells to the regulation by Tregs (96). For these reasons, it seems essential for therapeutic tolerance to become an effective intervention, to treat chronic inflammatory diseases in an early phase of the disease.

Initial clinical studies in patients with autoimmune diseases with a recent diagnosis using peptides derived from HSP have shown that the treatment is safe and also the possibility to skew the pro-inflammatory profile of pathogenic T-cell clones has been noted [reviewed in Ref. (97–99)]. For example, dnaJP1 is a 15 mer peptide, derived from bacterial HSP40, that shares homology with the “shared epitope” sequence present in some HLA-class II molecules associated with RA, which confers susceptibility to develop the disease. The peptide was identified as a pro-inflammatory T cell epitope in patients with active RA (100, 101), and authors hypothesized that it could be involved in the amplification of the inflammation due to the loss of regulatory mechanisms. In the double-blind, placebo-controlled phase II trial, dnaJP1 was administrated to active RA patients (<5 years of diagnosis) with the aim of inducing mucosal tolerance to this pro-inflammatory epitope. The treatment consisted in the administration of 25 mg of the peptide by oral route daily for 6 months. A decreased percentage of CD3^+^ T cells producing TNF-α in response to restimulation *in vitro* with dnaJP1 was observed in patients treated with the peptide but not with placebo. A trend toward increased levels of IL-10 was seen only in clinical responders. The expression of certain molecules associated with the downregulation of the immune response before the therapy was found necessary for successfully tolerization using this peptide. Finally, authors reported significant differences between treatment groups on day 140 for both American College of Rheumatology (ACR)20 and ACR50 responses (102).

More recently, a randomized placebo-controlled double-blind phase I/IIA trial was performed in patients with unresponsive active RA using binding immunoglobulin protein (BiP). BiP is an endoplasmic reticulum resident chaperone and stress protein with strong tolerogenic effects in the collagen-induced arthritis model (103). A single i.v. infusion of BiP (1, 5, or 15 mg) was well tolerated. The efficacy in this study was confounded by a high clinical response in the placebo group. However, at the end of the follow up period (12 weeks), remission was only achieved by some patients receiving 5 and 15 mg of BiP. Decreased C-reactive protein levels, VEGF, and IL-8 were decreased in patients receiving BiP compared with placebo at that time point (104). Finally, it was concluded that a large study is required to find the optimum dose and frequency of BiP administration.

Antigen-specific tolerance using HSP-derived peptides has also been explored in T1D. DiaPep277 is a 24-amino-acid peptide derived from the 437–460 sequence of HSP60. The treatment of newly diagnosed diabetic patients with DiaPep277 was well tolerated. In some patients, the treatment may delay the loss of the C-peptide production thereby decreasing the demand for exogenous insulin when compared with placebo groups in phase I and II clinical trials (105). The study of the T-cell populations of patients treated with DiaPep277 but not with placebo showed a shift toward a Th2 phenotype characterized by reduced levels of IFN-γ and increased expression of IL-4, IL-10, and IL-13 (106).

In general, immunological effects often correlate with a trend to clinical efficacy compared with placebo groups. However, the clinical efficacy has been less than expected. Therefore, it may turn out necessary to combine various strategies. For example, anti-TNF-α drugs may be combined with HSP peptide-based vaccination to have a synergic effect of inhibition of inflammation in combination with a Treg inducing strategy. A recently probed intervention was utilizing autologous tolerogenic dendritic cells (DCs) loaded with (autologous) synovial fluids in patients with progressive forms of RA (107). This first phase clinical trial showed the safety and the attainability of the approach. Although in some patients also a beneficial clinical effect was noted, it now seems needed to repeat such an intervention in patients with less advanced forms of the disease. Since it will be less practical to obtain synovial fluids from such patients, an attractive alternative possibility will be the use of HSP70 peptide B29. Besides the fact that B29 has shown a capacity to induce HSP70-specific Tregs, an additional advantage of using a well-defined antigen, such as B29, is that this will provide an opportunity to monitor the effect of the intervention precisely at the level of peptide-specific T cells. A clinical trial exploring the effect of B29 in combination with tolerogenic DCs in patients with RA is under development.

Although clearly in its infancy, therapeutic tolerance is expected to become a reality. In the case of RA, therapeutic progress has been significant until now. From the first pain killers, such as aspirin that was already available in the end of the nineteenth century, gold preparations since the 30s of the previous century, prednisone since World War II and biologics more recently, a very significant progress was made. The typical anatomical joint aberrations as they were seen frequently in RA patients are fully avoidable these days. Nonetheless, none of these interventions leads to cure. When therapy is halted, disease returns. Knowing this, the real challenge for the coming years will be the development of interventions that lead to a permanent remission based on regained self-tolerance. Given their supposed physiological role as targets for T cell regulation, HSPs may provide us possibly with the means to achieve true therapeutic tolerance.

## Author Contributions

AB, QL, and WE did the writing. FB, AS, and VR were involved with the design of the paper.

## Conflict of Interest Statement

The authors declare that the research was conducted in the absence of any commercial or financial relationships that could be construed as a potential conflict of interest.
